# Influenza-associated pneumonia as reference to assess seriousness of coronavirus disease (COVID-19)

**DOI:** 10.2807/1560-7917.ES.2020.25.11.2000258

**Published:** 2020-03-19

**Authors:** Kristin Tolksdorf, Silke Buda, Ekkehard Schuler, Lothar H Wieler, Walter Haas

**Affiliations:** 1 Robert Koch Institute, Berlin, Germany; 2 Helios Kliniken GmbH, Berlin, Germany

**Keywords:** SARS-CoV-2, COVID-19, seriousness of disease, hospital, pneumonia

## Abstract

Information on severity of coronavirus disease (COVID-19) (transmissibility, disease seriousness, impact) is crucial for preparation of healthcare sectors. We present a simple approach to assess disease seriousness, creating a reference cohort of pneumonia patients from sentinel hospitals. First comparisons exposed a higher rate of COVID-19 patients requiring ventilation. There were more case fatalities among COVID-19 patients without comorbidities than in the reference cohort. Hospitals should prepare for high utilisation of ventilation and intensive care resources.

As severe acute respiratory syndrome coronavirus 2 (SARS-CoV-2) spreads globally, crucial information on severity of the epidemic is needed. According to the World Health Organization (WHO) guideline on Pandemic Influenza Severity Assessment, severity indicators would be transmissibility, disease seriousness and impact [1]. Transmissibility reflects the movement of the virus, which is influenced by the dynamics of the spread, the R_0_ and the susceptibility of the exposed population. So far, several estimates on R_0_ exist, ranging between 1.4 to 6.49, which indicate a higher transmissibility than seasonal influenza, and even higher than SARS-CoV [2]. Impact reflects the impact on the healthcare sector, such as capacity utilisation of general practitioners, hospitals and public health authorities, and on society. Given the current data, the impact in countries other than China is hard to assess, although potentially high [3,4]. Disease seriousness reflects the extent of individual sickness including clinical symptoms, complications and outcomes. Recent publications from the area first affected in China (the city of Wuhan in the province of Hubei) offer valuable, although preliminary, data such as the proportion of hospitalised coronavirus disease (COVID-19) patients treated in intensive care units (ICU), ventilated or deceased. These are important parameters for the assessment of individual disease seriousness. However, a challenge is to apply these data to the situation in Europe, given the different population structures and comorbidities.

We want to introduce the concept of using syndromic surveillance data to assess disease seriousness of COVID-19, directly relating the results from clinical studies and case series on COVID-19 pneumonia patients to the situation as it is observed in pneumonia patients at the beginning of seasonal influenza epidemics. 

## Concept 

With a simple approach, we give a preliminary assessment of individual seriousness of COVID-19 using well-described case series of hospitalised COVID-19 pneumonia patients from the cities of Wuhan, Beijing, Shenzhen and the provinces of Hubei and Zhejiang [5-12]. We defined a reference group from a well-known setting: in 73 German sentinel hospitals, we extracted the data of all inpatients diagnosed with pneumonia (International Classification of Diseases, 10th revision codes J12-J18, primary diagnosis [13]) that were admitted during three consecutive weeks, after the start and before the peak of the influenza epidemic in the years 2015 to 2019. We compared severity parameters that were described for COVID-19 patients (acute respiratory distress syndrome, ventilation, intensive care, case fatality) with those from the German sentinel system. Furthermore, we stratified parameters by potential risk groups such as age, sex and chronic comorbidities. We also compared outcomes and risk factors for critically ill patients (i.e. received intensive care and ventilation) [14]. As the Chinese population is younger, with a larger proportion of males compared with the German population, we applied weights and provided crude and adjusted proportions for the sentinel pneumonia patients (SPP) [15]. This approach, which can be used by other countries using syndromic surveillance, attempts to replicate the uncertainty of initial results for the new coronavirus.

## Demographics, comorbidities and outcomes

Crude and adjusted values of mean and median age were lower in all COVID-19 case series compared with SPP (Table). The proportion of females differed between the case series, ranging from 32% in Wuhan to 59% in Zhejiang. The adjusted SPP showed a range from 39% to 42% in the proportions of females throughout the 5 years under consideration. The overall proportion of comorbidities was much smaller in the COVID-19 case series (range: 20–51%) than in German patients, where the proportion ranged from 70% to 77%, depending on the year. Hypertension and diabetes were the most important chronic comorbidities both among COVID-19 patients and among SPP. However, there were fewer patients with chronic obstructive pulmonary disease (COPD) or renal disease among COVID-19 patients than among SPP.

**Table:** Epidemiological and clinical characteristics of sentinel pneumonia patients, crude and adjusted for age and sex, and COVID-19 case series

Characteristics	SPP, crude	SPP, adjusted	COVID-19							
	Weeks 3–5, 2015–19	Weeks 3–5, 2015–19	20 days	28 days	31 days	26 days	22 days	30 days	17 days	23 days
			Wuhan [5]	Wuhan [6]	Wuhan [7]	Hubei [10]	Beijing [8]	Shenzhen [9]	Zhejiang [11]	Zhejiang [12]
Number	5,829	NA	99	138	82	137	262	298	62	91
Mean age in years (range)	73 (71–75)	63 (61–66)	55.5	NA	NA	55	NA	NA	NA	NA
Median age in years (range)	77 (76–78)	67 (65–69)	NA	56	NA	57	47.5	47	41	50
Female, % (range)	45 (44–45)	41 (39–42)	32	46	NA	56	52	50	44	59
Any chronic disease, % (range)	82 (80–85)	74 (70–77)	51	46	NA	20	NA	32	32	NA
Hypertension, % (range)	58 (56–62)	49 (46–53)	NA	31	NA	10	NA	13	8	16
Endocrine condition/diabetes, % (range)	28 (26–30)	24 (22–26)	13	14	NA	10	NA	6	2	9
COPD, % (range)	20 (19–22)	20 (18–22)	NA	3	NA	2	NA	NA	2	NA
Cancer, % (range)	7 (6–8)	8 (7–9)	1	7	NA	2	NA	1	NA	NA
Renal disease, % (range)	37 (34–40)	27 (24–29)	NA	3	NA	NA	NA	NA	2	NA
Liver disease, % (range)	4 (3–4)	5 (3–6)	NA	3	NA	NA	NA	3	11	NA
Of total: ARDS, % (range)	1 (0–1)	1 (0–1)	17	20	NA	NA	NA	4	2	NA
Of total: ventilated, % (range)	8 (7–10)	9 (8–11)	20	23	NA	25	NA	11	2	NA
Of total: ICU, % (range)	19 (18–21)	20 (18–22)	23	26	NA	NA	NA	11	2	10
Of ICU: male, % (range)	58 (55–60)	61 (56–66)	NA	61	NA	NA	NA	NA	NA	NA
Of ICU: median age in years (range)	76 (74–78)	67 (64–68)	NA	66	NA	NA	NA	NA	NA	NA
Of ICU: without chronic, % (range)	10 (7–12)	16 (10–19)	NA	28	NA	NA	NA	NA	NA	NA
Of total: deceased, % (range)	9 (8–9)	6 (5–6)	11	4	6	12	1	0	0	0
Of deceased: median age, in years (range)	83 (82–84)	78 (76–79)	NA	NA	72.5	NA	NA	NA	NA	NA
Of deceased: < 60 years, % (range)	5 (2–9)	13 (7–22)	36	NA	20	NA	30	NA	NA	NA
Of deceased: without chronic disease, % (range)	13 (13–15)	13 (12–14)	18	NA	23	NA	30	NA	NA	NA
Still hospitalised, %			58	62	NA	56	82	77	98	66

ARDS: acute respiratory distress syndrome; COPD: chronic obstructive pulmonary disease; COVID-19: coronavirus disease; ICU: intensive care unit; NA: not available; SPP: sentinel pneumonia patients.

## Intensive care unit

**Figure 1:**
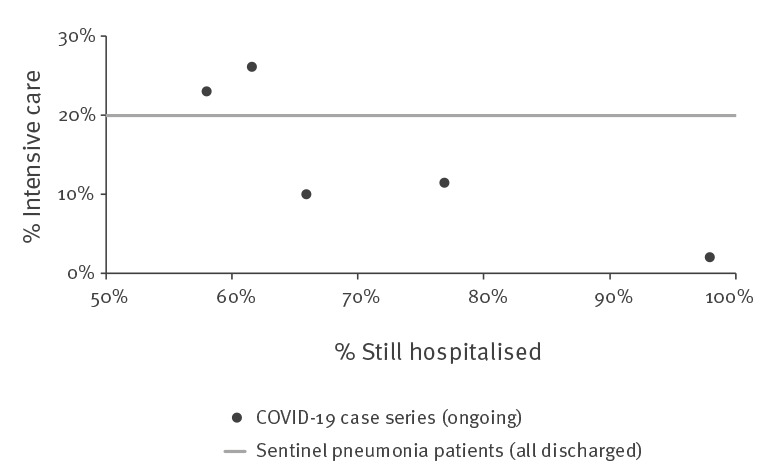
Reported proportions of patients in intensive care and proportions of patients who were still hospitalised, five COVID-19 case series, China, January–February 2020 (n = 688)

COVID-19: coronavirus disease; ICU: intensive care unit.

The proportion of ICU patients in the SPP was 20%, ranging from 18% in 2016 to 22% in 2019. This was the same scale as described in two case series from Wuhan in the province of Hubei [5,6]. However, the case series from provinces outside of Hubei reported only 11%, 10% and 2% (one case) of ICU patients among COVID-19 cases, which is remarkably lower [9,11,12]. This may be attributed to the preliminary character of the outcome, as more than half of the cases were still hospitalised in these case series (Table and Figure 1).

Grey line: proportion of ICU in pneumonia sentinel patients. Data from five COVID-19 series [5,6,9,11,12].

Among ICU patients, the proportion of males and the median age were strikingly similar between COVID-19 (61% and 66 years) and SPP (61% and 67 years) [6]. However, the proportions of chronic comorbidities were different: 28% of COVID-19 patients treated on the ICU did not have any reported comorbidity, whereas only 16% of German ICU patients were without comorbidities (Table).

### Ventilation

**Figure 2:**
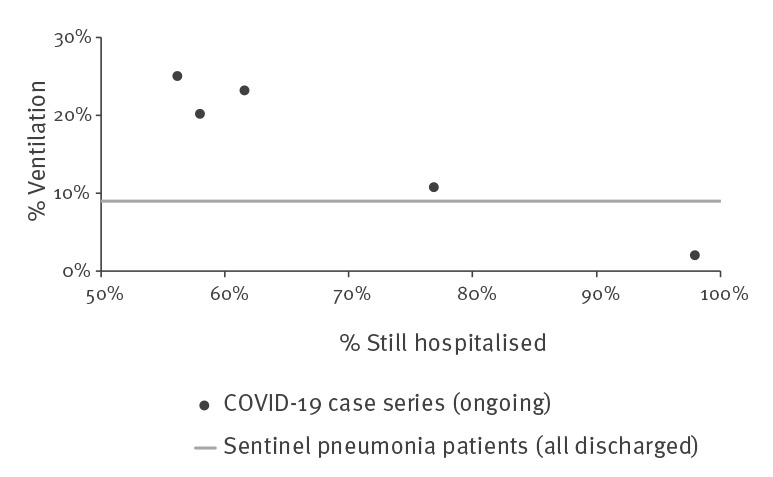
Reported proportions of patients on ventilation and proportions of patients who were still hospitalised, five COVID-19 case series, China, January–February 2020 (n = 734)

Three case series from the province Hubei described a high rate of cases who needed ventilation (20%, 23% and 25%) [5,6,10]. German pneumonia patients had a two- to threefold lower ventilation rate of 9%. The two case series from Shenzhen and the province Zhejiang also reported much lower ventilation rates of, respectively, 11% and 2% [9,11] (Figure 2). The median duration of ventilation was 9 days (interquartile range: 7–19, n = 13) for non-invasive and 17 days (interquartile range: 12–19, n = 4) for invasive ventilation in the COVID-19 case series described by Chen et al. [5]. In contrast, the median ventilation duration (invasive and non-invasive) in SPP was only 2 days (interquartile range: 1–4, n = 303). The high ventilation rates and the long duration of ventilation (data from one case series) can be attributed to a high rate of acute respiratory distress syndrome (ARDS) among COVID-19 patients. Again, the difference between Hubei (17–20% ARDS) and outside of Hubei (2–4% ARDS) is striking. However, ARDS was observed in only 1% of SPP.

COVID-19: coronavirus disease.

Grey line: proportion of ventilation in pneumonia sentinel patients. Data from five COVID-19 series [5,6,9-11].

### Case fatality

**Figure 3:**
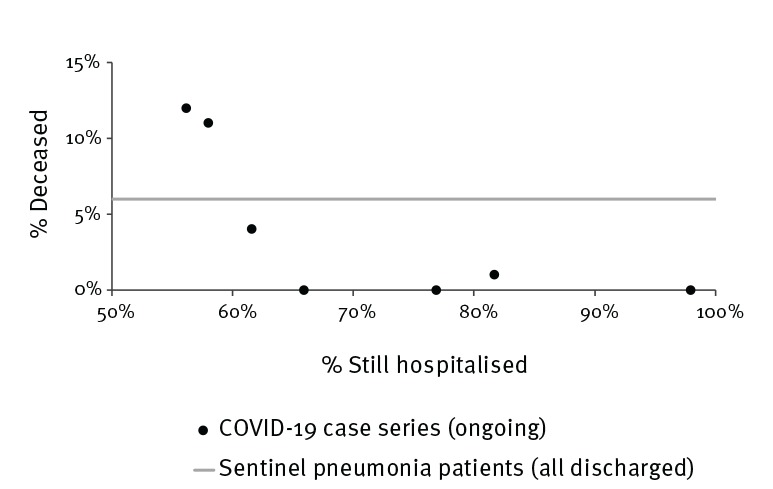
Reported proportions of deceased and corresponding proportions of patients that were still hospitalised, seven COVID-19 case series, China, January–February 2020 (n = 1,087)

COVID-19: coronavirus disease; SARS-CoV: severe acute respiratory syndrome coronavirus.

The observed case fatality ratios in the described case series from Hubei (including the city of Wuhan) ranged from 4% to 12%. Most case series outside Hubei did not report fatalities. One case series had 1% case fatality. However, more than two thirds of the cases reported from outside Hubei were still hospitalised at the reporting dates. The case fatality ratio among SPP was 6%, which is within the range of the reported COVID-19 case fatalities (Figure 3).

Grey line: proportion of deceased in pneumonia sentinel patients. Data from five COVID-19 series [5,6,8-12].

A study by Yang et al. described clinical courses and outcomes of 52 critically ill patients in a hospital in Wuhan [14]. Among 25 critically ill COVID-19 patients younger than 60 years, 12 died within 28 days after admission to the ICU. Of 31 patients without chronic illnesses, 15 died, which is a comparable proportion of almost half.

In the sentinel hospitals, we identified 462 pneumonia patients who were critically ill (i.e. received intensive care and ventilation). Of those patients, 92 were younger than 60 years, of whom 12 (13%) died. In addition, 18 of the critically ill pneumonia patients were without chronic preconditions and four of them died.

## Discussion

Considering the 5 years from 2015 to 2019, the proportion of severe cases requiring intensive care and the case fatality ratios were strikingly similar among COVID-19 and German pneumonia patients. However, based on these data, COVID-19 may affect a younger cohort, which seems not fully explained by the different age structure of the Chinese population. As reported by the China Centre for Disease Control and Prevention, there seems to be a higher risk for severe disease in older ages and in patients with chronic illnesses [16]. But severity in younger adults below 60 years and in patients without chronic preconditions appears to be higher in COVID-19 patients than in pneumonia patients usually seen during the influenza season. The rate of ARDS and of patients requiring ventilation was markedly higher among COVID-19 patients with much longer duration of ventilation, based in the data from one study. During the influenza pandemic 2009 however, specialised German hospitals reported similarly long ventilation duration times [17].

The case series from outside Hubei report much milder symptoms than those reported from Hubei, especially from Wuhan, which may be due to early admittance of contact persons and of suspected cases with only mild symptoms. In addition, the large impact on the health system in the initially affected province resulted in insufficient healthcare resources [3]. This may have inhibited adequate treatment as the strikingly higher case fatality indicates [18]. However, all COVID-19 case series were still open with more than half of the cases hospitalised at reporting date. It is known that COVID-19 cases can have a prolonged course with many fatalities occurring 3 weeks after symptom onset [19]. Case fatality rates from these COVID-19 case series can therefore only be seen as preliminary.

Moreover, variations in health systems that could result in over- or underdiagnosis of chronic comorbidities such as COPD [20,21] are unaccounted for and may contribute to some of the observed differences between COVID-19 and SPP.

## Conclusion

Our approach is flexible enough to create reference cohorts, which will allow estimation of COVID-19 severity using known characteristics and outcomes. As more data on European cases are published, the system can be applied to cases with a more comparable background. First comparisons expose the high rate of patients requiring ventilation over prolonged time periods, thus hospital resources may be in higher demand of ventilation supply than usual. In fact, Liu et al. suggests that early non-invasive mechanical ventilation can promote positive outcomes [9]. This can only be implemented if hospitals prepare for high utilisation of ventilation and intensive care resources. Although fatalities occur mostly among elderly people with chronic comorbidities, serious disease progressions do also occur among younger, healthy patients and more often than would be expected from the experience during influenza epidemics.

## Editorial note

This article was published as an e-Alert on 16 March 2020.
